# Midterm outcomes of revision biceps tendon augmentation without tear completion for high-grade partial articular-sided supraspinatus retear

**DOI:** 10.1016/j.jseint.2025.101612

**Published:** 2025-12-29

**Authors:** Young Jun Kim, Jin-Kyu Kang, Hwang-Young Yoo, Bo-Sung Kim, Jong-Hun Ji

**Affiliations:** a Department of Orthopedic Surgery, Incheon Sejong Hospital, Incheon, Republic of Korea; b Department of Orthopedic Surgery, Daejeon St. Mary's Hospital, The Catholic University of Korea, Daejeon, Republic of Korea

**Keywords:** Rotator cuff, Partial-thickness tear, Articular-sided retear, Transtendon repair, Revision surgery, Biceps tendon augmentation, Magnetic resonance imaging

## Abstract

**Background:**

To evaluate the midterm clinical and radiological outcomes of revision biceps tendon augmentation without tear completion for high-grade partial articular-sided supraspinatus retear (PASR) lesions, which present challenges due to poor tendon quality.

**Methods:**

From January 2014 to May 2023, a total of 22 patients (mean age: 57.2 ± 13.6 years, 8 males and 14 females) who underwent revision cuff repair with biceps tendon augmentation without tear completion for PASR lesions following arthroscopic rotator cuff repair were enrolled in our study. Clinical and radiological outcomes, including tendon integrity (Sugaya classification), were evaluated at the last follow-up.

**Results:**

Previous rotator cuff tears included 10 full-thickness rotator cuff tears and 12 partial-thickness rotator cuff tears. The mean interval between the primary and revision surgeries was 5.9 ± 6.0 years (range, 0.4-14 years). Significant improvements were observed in all clinical outcome scores at the final follow-up. The mean visual analog scale for pain improved from 3.8 preoperatively to 0.6 postoperatively. American Shoulder and Elbow Surgeons scores improved from 64 to 94, University of California, Los Angeles shoulder scores from 22 to 33, Simple Shoulder Test scores from 7 to 11, and Korean Shoulder Scoring System scores from 71 to 94 (all *P* values <.001). Follow-up magnetic resonance imaging demonstrated good tendon integrity, with Sugaya type I in 6 cases, type II in 15 cases, and type IV in 1 case. Mean tendon thickness increased from 4.6 mm to 7.4 mm. There were no retears, and no progression of fatty infiltration or muscle atrophy was observed.

**Conclusion:**

High-grade PASR lesions following arthroscopic rotator cuff repair are uncommon and challenging due to poor tendon quality. Revision biceps tendon augmentation without tear completion appears to be a reliable surgical option for PASR lesions, yielding favorable midterm functional and radiological outcomes.

Retears following primary transtendon repair of high-grade partial-thickness rotator cuff tears or suture bridge repair of full-thickness rotator cuff tears (FTRCTs) are challenging due to poor tendon quality, particularly in active elderly patients. Articular-sided retears after suture bridge repair of FTRCT are relatively rare. Yamakado reported that incomplete healing (deep-layer retraction pattern) was observed only in medial-based single-row patients.^27^ Moreover, poor tendon quality (thin and fragile) and adhesive scar tissue from prior repairs make articular-sided retear repair without tear completion particularly difficult. We term these lesions high-grade partial articular-sided supraspinatus retears (PASR). PASR lesions following primary transtendon or suture bridge repair are rare and challenging to treat due to poor tendon quality. Some patients with PASR lesions experience persistent or refractory chronic shoulder pain and, having undergone prior rotator cuff repair (transtendon or suture bridge), are often reluctant to pursue further revision surgery. Many manage their pain with conservative treatments, such as medication or injections.

These high-grade PASR lesions often respond poorly to conservative treatments and are difficult to treat due to poor tendon quality and scar contracture. The transtendon suture-bridge technique for high-grade partial articular supraspinatus tendon avulsion (PASTA) lesions has shown satisfactory clinical outcomes.^10^^,^^11^ Both arthroscopic transtendon repairs and tear completion (take-down) with rotator cuff repair appear to be effective operative techniques, with the rate of good to excellent results ranging from 86% to 94.1%.^11^^,^^20^^,^^21^ However, residual shoulder pain is frequently reported after transtendon repair of high-grade PASTA lesions.^2^^,^^19^ Transtendon repair often fails to restore the articular side of the rotator cuff tendon to its anatomic footprint, potentially causing tension mismatch between the bursal and articular layers.

Retears after primary transtendon repair of high-grade PASTA lesions or suture bridge repair of FTRCT often lead to scar adhesion. In revision surgery, tear completion and débridement of poor-quality tendon can result in a larger cuff defect. The retear rate following revision rotator cuff repair is reportedly higher than that of primary repair. Several treatment options exist for PASR lesions, including arthroscopic débridement, transtendon repair, tear completion and repair, or patch augmentation.^13^^,^^15^^,^^17^^,^^22^

Arthroscopic débridement or revision transtendon repair often fails to restore normal cuff tendon integrity due to thin and fragile tendon tissue. Patch augmentation is limited by its high technical demands and associated financial burden. Tear completion with cuff repair is the most commonly used method for failed in situ (transtendon) repairs, as it is often considered the treatment of choice. However, tear completion disrupts the intact rotator cuff footprint, leading to a larger cuff defect and suboptimal healing compared to the natural enthesis.^1^^,^^12^

In contrast, revision biceps tendon augmentation without tear completion (transtendon suture bridge repair with biceps tendon augmentation) preserves the intact bursal side cuff attachment and has demonstrated satisfactory clinical outcomes.^10^ Additionally, transtendon repair offers superior biomechanical properties, including better footprint coverage, reduced gapping, and greater failure strength compared to tear completion and repair.^18^^,^^26^ Moreover, biceps tendon augmentation reinforces high-grade PASTA lesions by incorporating the tenotomized biceps tendon into the torn articular side cuff defect, providing an autologous collagen matrix.^10^^,^^23^^,^^28^ This technique effectively provides an autologous collagen matrix via the biceps tendon, aiding in the restoration of poor-quality tendon in high-grade PASR lesions.

The purpose of this study was to evaluate the clinical and radiological outcomes of the revision biceps tendon augmentation without tear completion in PASR lesions. We hypothesized that this technique would provide a collagen matrix via the biceps tendon, restore sufficient tendon thickness, and achieve favorable outcomes without complications.

## Methods

This retrospective study included 22 consecutive patients who underwent revision biceps tendon augmentation for high-grade PASR lesions, performed by a single senior surgeon between January 2014 and May 2023. The minimum follow-up period was 2 years. The mean age was 57.2 ± 13.6 years (range, 47-71 years). Eight patients were male, and 14 were female. Four patients had a trauma history. The average time to revision surgery was 5.9 ± 6.0 years (range, 0.4-14 years) from the index procedure (Table I).

**Table I:** Demographics of patients with high-grade partial articular-sided supraspinatus tendon retear (PASR) lesions.

Variable	Value
Age (yr)	57.2 ± 13.6 (47-71)
Sex (male/female)	8/14
Body mass index (BMI, kg/m^2^)	29.2 ± 13.0 (20.5-32.9)
Symptom duration prior to revision (yr)	5.9 ± 6.0 (0.4-14)
Dominant arm (right/left)	21/1
Operative arm (right/left)	14/8
History of trauma (%)	4 (18.2%)

**Figure 1:**
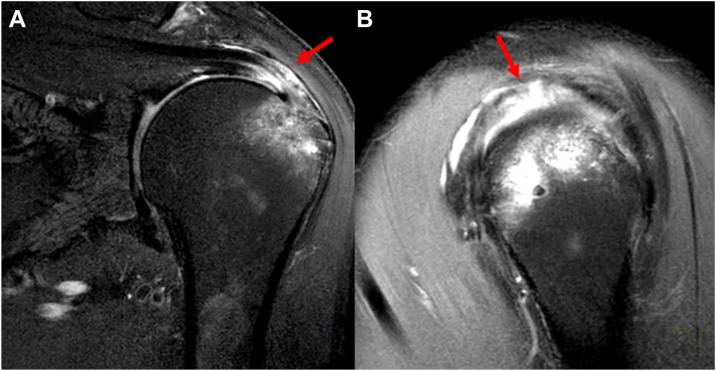
Preoperative magnetic resonance imaging (MRI) identifying a high-grade partial articular-sided supraspinatus retear (PASR). (**A**) A coronal T2-weighted fat-suppressed image reveals the PASR lesion. The *red arrow* indicates abnormal high signal intensity along the articular side of the supraspinatus tendon, while the bursal side remains intact. (**B**) A sagittal oblique view confirms the extent of the articular-sided defect (*red arrow*), clearly distinguishing it from a full-thickness tear. These multiplanar images provided a precise assessment of the high-grade retear.

The inclusion criteria consisted of symptomatic patients with high-grade PASR lesions following primary transtendon repair of high-grade PASTA lesions or suture bridge repair of FTRCTs. All patients experienced persistent shoulder discomfort after primary rotator cuff repair, with symptoms persisting or worsening despite at least six months of conservative treatment. Magnetic resonance imaging (MRI) confirmed the presence of high-grade PASR lesions. PASR lesions were confirmed using sagittal and coronal T2 fat-suppressed MRI scans, ensuring no full-thickness cuff tears (Fig. 1). Blinded one expert musculoskeletal radiologist diagnosed PASR lesions based on increased signal intensity between the articular side of the supraspinatus tendon and the greater tuberosity (medial footprint area), with an intact bursal side.

The initial diagnosis of PASR lesions was based on clinical symptoms and MRI findings and was subsequently confirmed through arthroscopic evaluation. Arthroscopic confirmation involved visual assessment of the exposed articular-side footprint and probing. Patients with high-grade PASR lesions (Ellman's Grade II or III [>3 mm] with poor tendon quality) and an intact biceps tendon were included. Exclusion criteria included complete biceps tendon tears, concomitant full-thickness subscapularis tendon tears, or glenohumeral arthrosis. Low-grade partial tears (Ellman's Grade I [<3 mm]) were excluded, as they underwent only arthroscopic débridement.

**Figure 2:**
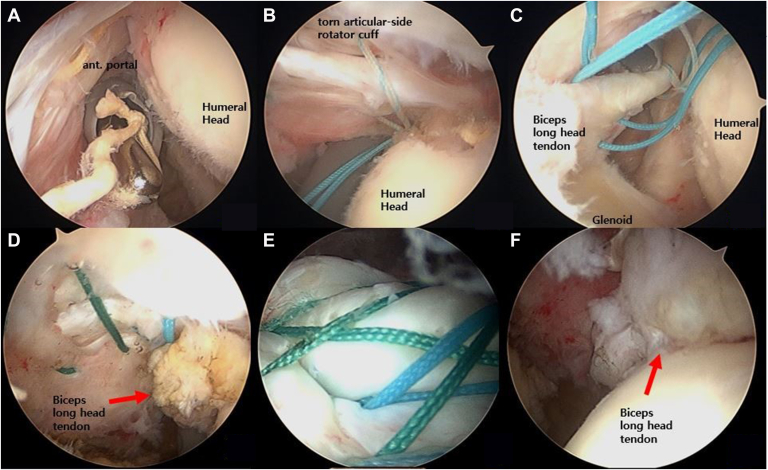
Surgical techniques for revision rotator cuff repair with biceps tendon augmentation without tear completion in PASR lesions. (**A**) A torn articular-side rotator cuff with poor tendon quality is shown, and the exposed threads from a previously placed suture anchor are removed. (**B**) After preparing the exposed footprint, new anchors are inserted, and the suture threads are passed through the rotator cuff. (**C**) Sutures are sequentially passed along the length of the biceps tendon using a Bird Beak Suture Passer. (**D**) The final positioning of the biceps tendon within the torn rotator cuff defect is achieved, with the sutures tightened to ensure proper abutment between the rotator cuff tendon and the biceps tendon. (**E** and **F**) In the subacromial space, 4 strands of the suture bridge rotator cuff repair are securely tied at the footprint, ensuring firm compression of the biceps tendon (*arrow*) and rotator cuff tissue in the glenohumeral joint. *PASR*, partial articular-sided supraspinatus retear.

**Figure 3:**
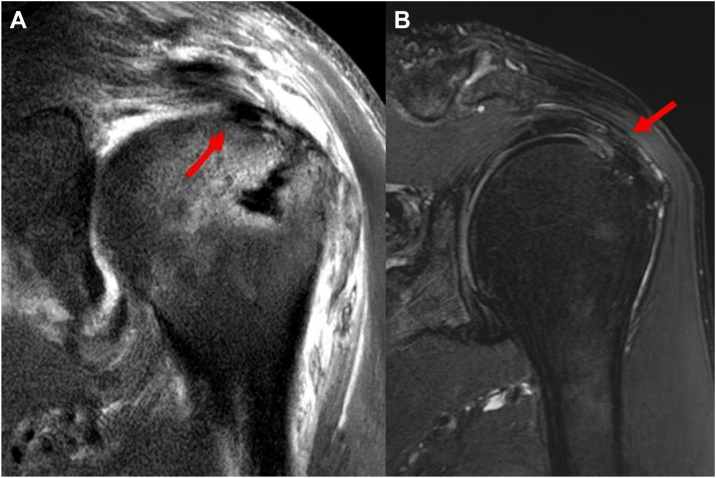
Postoperative MRI outcomes of the same patient showing successful revision repair. (**A**) A postoperative coronal T2-weighted image demonstrates the augmented biceps tendon (*red arrow*) well-seated beneath the supraspinatus tendon, effectively filling the articular-sided defect. (**B**) A postoperative sagittal oblique view shows the restored continuity and structural integrity of the rotator cuff with the biceps augmentation (*red arrow*). *MRI*, magnetic resonance imaging.

Retrospectively collected data were evaluated, and all patients' clinical and radiologic information was reviewed. Outcome assessments included shoulder range of motion (ROM), clinical scores (American Shoulder and Elbow Surgeons score [ASES], University of California, Los Angeles shoulder score [UCLA], Korean Shoulder Scoring System [KSS], Simple Shoulder Test [SST], and visual analog scale), and complications such as infection, Popeye deformity, and retears. All patients underwent shoulder arthroscopy in the lateral decubitus position. Arthroscopic examination confirmed a retear (PASR lesions) with an intact bursal side cuff and biceps tendon. Arthroscopic transtendon suture bridge repair with biceps tendon augmentation was performed for PASR lesions (Fig. 2). Postoperative MRI evaluations were conducted at a mean of 9 months after surgery. Coronal and sagittal T2-weighted MRI scans assessed tendon integrity (using the Sugaya classification), biceps tendon incorporation, fatty infiltration, and muscle atrophy of the supraspinatus tendon (Fig. 3).

Postoperative T2-weighted fat-suppressed oblique coronal MRI images were used to assess and compare repaired tendon thickness at the preoperative and final follow-up stages. Thickness was measured as the vertical distance from the midpoint of the footprint around the greater tuberosity. Typically, the repaired tendon thickness was measured on the third or fourth image slice from the long head of the biceps tendon. In conventional MRI, a 3 mm slice thickness was used, with measurements typically taken 9-12 mm posterior to the long head of the biceps tendon.

### Surgical procedure

Under general anesthesia with an interscalene block, all patients were operated on in the lateral decubitus position (Fig. 2). In the glenohumeral joint, high-grade PASR lesions (Ellman's Grade II or III with poor tendon quality) and an intact biceps tendon were identified. The torn edges of the retear (PASR lesions) were gently débrided to facilitate tissue mobilization. During the procedure, exposed threads from previously placed suture anchors were removed, and all adhesions to the surrounding tissue were released (Fig. 2A). Initially, mobilization was attempted to bring the torn articular-side rotator cuff to the exposed footprint area. Using a shaver or burr, the exposed tuberosity bone of the torn supraspinatus tendon was débrided, and a suture anchor was inserted at the footprint, just lateral to the articular margin of the humeral head (Fig. 2B). All suture threads from the anchor were retrieved through the anterior portal.

Each strand of the suture anchor was then sequentially passed through the biceps tendon (from posterior to anterior, from its insertion on the glenoid to the distal end) using a Bird Beak Suture Passer (Arthrex Inc., Naples, FL, USA) through the anterior portal (Fig. 2C). An 18-gauge spinal needle was inserted percutaneously at the rotator cuff tear margin, and a nonabsorbable suture was used to shuttle the suture limbs from posterior to anterior. Consequently, the 4 strands retrieved in the anterior portal, which had previously penetrated the biceps tendon, were passed through the cuff. Next, the insertion of the biceps tendon was cut using an ArthroWand (ArthroCare, Austin, TX, USA), and its abutment within the defect on the articular side was confirmed by pulling the threads (Fig. 2D). The arthroscope was then moved to the subacromial space, where 2-4 sliding knots were tied using one double-loaded, one triple-loaded nonmetallic anchor, or 2 double-loaded soft anchors depending on the tear size, completing the medial row repair. Additional compression of the cuff tissue to its footprint (lateral row repair) was achieved using lateral row anchors with the suture bridge technique (Fig. 2E). All anchors used in this study were nonmetallic to minimize artifacts regarding postoperative MRI evaluation.

Finally, the arthroscope was repositioned into the glenohumeral joint to confirm the integrity of the repaired cuff tendon, as well as the attachment and incorporation of the biceps tendon beneath the torn articular-side rotator cuff (Fig. 2F).

Postoperatively, patients were immobilized in a sling for 6 weeks. Passive ROM exercises, such as pendulum exercises, were initiated immediately and continued for 6 weeks after surgery. Active ROM exercises began at 6 weeks postoperatively, and strengthening exercises were permitted at 12 weeks.

### Statistics

Statistical analysis was performed using SPSS 20 (IBM Corp., Armonk, NY, USA), with a significance threshold of *P* < .05. Continuous data, including preoperative and postoperative clinical scores and ROM, were analyzed using the Student's *t*-test or Mann-Whitney U-test, and categorical data were assessed with the chi-square test or Fisher's exact test. Internal rotation was evaluated based on the vertebral level reached by the thumb (using the Wilcoxon signed-rank test). Radiologic measurement reliability (Sugaya classification, retear) was assessed using Cohen's kappa statistics and the intraclass correlation coefficient, with agreement classified as excellent (κ > 0.75), fair to good (κ = 0.4-0.75), or poor (κ < 0.4). Intraobserver and interobserver reliability for radiologic assessments was excellent.

## Results

All patients in this study presented with persistent or refractory shoulder pain for a mean of 5.9 ± 6.0 years (range, 0.4-14 years) following their primary cuff repair. Through MRI and arthroscopy, we confirmed PASR lesions in all patients, and then revision repair (transtendon suture bridge repair with biceps tendon augmentation) was performed. The mean follow-up period was 2.0 ± 1.42 (1-6) years.

### Clinical outcomes

At the final follow-up, clinical scores and ROM were improved significantly in all patients compared to prerevision surgery status. All patients had improved refractory shoulder pain after revision surgery. Pain visual analog scale scores improved from preoperative 3.8 (3-5) to postoperative 0.6 (0-2); ASES scores from 64 to 94; UCLA scores from 22 to 33; SST scores from 7 to 11; KSS scores from 71 to 94 (all *P* value <.001). Preoperative clinical scores including ASES, UCLA, SST, and KSS improved significantly after surgery (*P* < .05). Significant improvements were observed in forward flexion (*P* = .009), abduction (*P* = .005), and internal rotation (*P* = .008), whereas external rotation showed no statistically significant improvement (*P* = .151) (Table II).

**Table II:** Clinical outcomes after the revision rotator cuff repair (transtendon suture bridge repair) with biceps augmentation in high-grade partial articular-sided supraspinatus tendon retear (PASR) lesions.

	Preoperative	Last follow-up	*P* value
VAS pain score (0-10)	3.8 ± 0.7 (3-5)	0.6 ± 0.6 (0-2)	<.001
Subjective Shoulder Value (SSV, %)	47.8 ± 8.0 (25-65)	92.9 ± 7.0 (80-100).	<.001
ASES score (0-100)	64.3 ± 16.7 (10-88)	93.5 ± 4.1 (86-100)	<.001
UCLA score (0-35)	21.8 ± 6.3 (12-33)	33.0 ± 1.4 (31-35)	<.001
KSS score (0-100)	70.8 ± 16.8 (40-89)	94.3 ± 2.2 (88-100)	<.001
SST score (0-12)	6.7 ± 2.3 (2-11)	11.2 ± 1.0 (9-12)	<.001
Range of motion (°**)**			
Forward flexion	144.0 ± 19.3	156.7 ± 9.5	.009
Abduction	142.6 ± 21.0	156.2 ± 9.6	.005
External rotation	23.3 ± 16.5	30.5 ± 12.4	.151
Internal rotation (average vertebral level)	T11 (T10 ± L3)	T9 (T7 ± T12)	.008

*VAS*, visual analog scale; *ASES*, American Shoulder and Elbow Surgeons score; *UCLA*, University of California, Los Angeles shoulder score; *KSS*, Korean Shoulder Scoring System; *SST*, Simple Shoulder Test.

Wilcoxon signed-rank test.

### Radiologic outcomes

At the postoperative MRI evaluation (mean 8 months), improved footprint coverage with biceps tendon augmentation and good tendon integrity with sufficient thickness were observed. Follow-up MRI findings demonstrated significantly increased supraspinatus tendon thickness, indicating successful incorporation of the biceps tendon to the PASR lesion (*P* < .001). These findings were consistent with adequate tendon thickness and healing, as classified by Sugaya type I or II.

**Figure 4:**
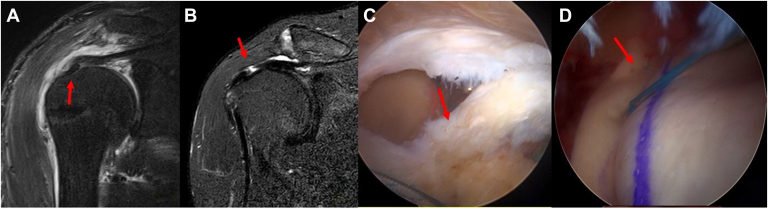
Re-revision rotator cuff repair of the partial articular side supraspinatus retear (PASR) lesion. (**A**) A revision rotator cuff repair with biceps tendon augmentation (*arrow*) without tear completion for PASR lesions was performed. (**B**) One year postoperatively, follow-up MRI demonstrated a full-thickness rotator cuff retear. (**C**) Arthroscopic evaluation confirmed a full-thickness rotator cuff tear measuring 1.5 × 1.5 cm. However, the previously augmented biceps tendon remained well incorporated into the rotator cuff footprint (*arrow*). (**D**) A rerevision bridging rotator cuff repair was subsequently performed using an acellular dermal matrix. *MRI*, magnetic resonance imaging.

Among the patients, 6 were classified as Sugaya type I, fifteen as Sugaya type II, and one as Sugaya type IV (Fig. 4). One patient, who presented with a Sugaya type IV finding (indicating a failed repair, classified as a type 2 retear), underwent rerevision surgery consisting of a bridging repair with an acellular dermal matrix.^4^ Even though in this patient, previous biceps tendon augmentation was well healed and preserved into the rotator cuff footprint (type 2 retear). MRI scans confirmed that the biceps tendon had effectively integrated into the repaired footprint, further contributing to the structural integrity of the rotator cuff. Follow-up MRI showed sufficient thickness (from 4.6 to 7.4 mm) without retears.

**Figure 5:**
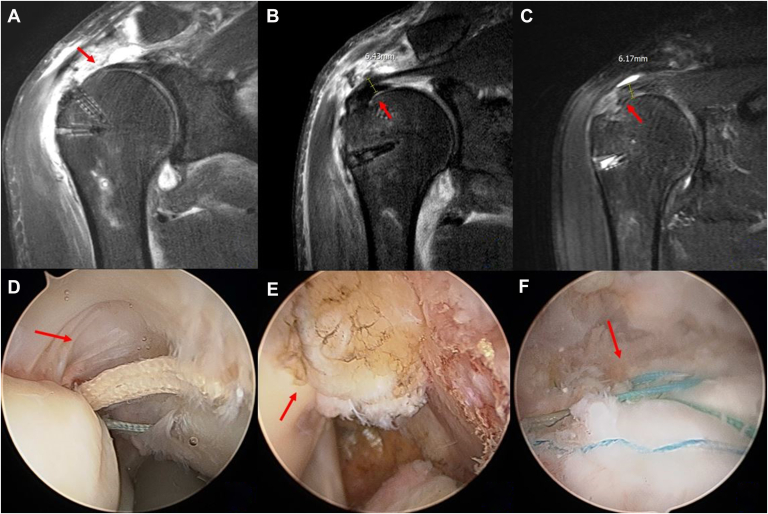
Tendon thickness and arthroscopic findings. MRI findings of the revision rotator cuff repair with biceps tendon augmentation without tear completion. (**A**) A coronal T2 fat-suppressed MRI scan of the supraspinatus tendon showed ill-defined contour and irregular intermediate T2 signal intensity change (*arrow*) in which was compatible with a PASR (partial articular-side supraspinatus retear) lesion. (**B**) A revision rotator cuff repair with biceps tendon augmentation (*arrow*) without tear completion for PASR lesions was performed. (**C**) 6 months postoperatively, follow-up MRI demonstrated a good incorporation of the augmented biceps tendon (*arrow*) into the native tendon. (**D**) Arthroscopic finding showed high-grade PASR lesion (*arrow*) and the bursal side of the supraspinatus tendon remained intact. (**E**) The augmented biceps tendon remained well incorporated into the rotator cuff footprint (*arrow*) in the glenohumeral joint. (**F**) In the subacromail space, transtendon suture bridge rotator cuff repair (*arrow*) was performed. *MRI*, magnetic resonance imaging.

These findings showed sufficient tendon thickness (Sugaya type I or II) without evidence of retear (Fig. 5). Further progression of fatty infiltration (mean grade: 2.0 to 2.0) and muscle atrophy (mean grade: 1.2 to 1.2) was not observed in the follow-up MRI evaluation (*P* > .05) (Table III).

**Table III:** Postoperative radiologic outcomes after transtendon suture bridge repair with biceps augmentation in high-grade partial articular-sided supraspinatus tendon retear (PASR) lesions.

	Preoperative	Postoperative	*P* value
Sugaya classification change	Sugaya type I in 6 cases, type II in 15 cases, and type IV in 1 case.		
Tendon thickness	3.3 ± 1.3 (2.0-8.4) mm	5.6 ± 2.2 (0.0-10.0) mm	<.05
Fatty infiltration	2 (1-3)	2 (1-3)	.577

## Discussion

The principal finding of this study is that revision surgery using autologous biceps tendon augmentation for PASR lesions resulted in significantly improved functional outcomes and increased tendon thickness at midterm follow-up. Follow-up MRI showed sufficient tendon thickness and preserved footprint coverage with biceps tendon augmentation, and only one case recorded retear. Clinical and radiological outcomes significantly improved compared to preoperative status.

Incomplete healing and poor tendon quality after primary repair often prompt surgeons to convert them into full-thickness tears, making revision repair more challenging. Especially, PASR lesions were rare following primary trans-tendon repair of high-grade PASTA lesions or suture bridge repair of full-thickness tears. This rarity is particularly notable because PASR lesions after these procedures are an uncommon finding for most arthroscopic surgeons. These PASR lesions are, sometimes, too subtle to diagnose clinically or radiologically. MRI findings of PASR lesions typically show ill-defined contours and heterogeneous signal intensity, making revision surgery without footprint damage (tear completion and repair) challenging. Also, residual shoulder pain is common after transtendon repair of high-grade PASTA lesions.^1^

Few studies have investigated revision PASR lesions without tear completion. Therefore, we compared our midterm outcomes with those reported for primary PASTA repairs in the literature. Castracini et al,^3^ in their series, used 2 techniques to repair PASTA lesions-transtendon repair and tear completion reported an average SST score of 10.1 (5-12) at midterm follow-up after repair, which is similar to our study. Their reported retear rate of 13.9% for transtendon repair is considerably higher than the 4.5% (1 of 22 patients) retear rate observed in our cohort. Plachel et al^16^ in their long-term study of grade II PASTA repair reported UCLA score, 31 ± 7 points; ASES score, 85 ± 24 points; SSV, 83% ± 21% which is similar to our findings. Radiologic evaluation in their study showed progression to a FTRCT in 6% of patients, and 60% of the patients showed persistent signs of partial tearing evaluated by MRI, which is higher than our study. Our study demonstrated satisfactory midterm clinical outcomes, with only one retear in a patient with thin, poor-quality rotator cuff tissue (type 2 retear).^4^

The retear rate after revision rotator cuff repair is reportedly as high as double that of primary repairs. After retear of conventional cuff repair, the common form of presentation is pain, weakness, and stiffness of the affected shoulder.^2^^,^^7^^,^^19^ Conventional transtendon repair often leads to tension mismatch and residual discomfort due to bulging of the bursal layer of the cuff. Our transtendon suture bridge repair with biceps augmentation mitigates these issues by reducing articular layer overtightening, minimizing tension mismatch, and enhancing compression to address bursal bulging.^9^^,^^13^ During the rotator cuff repair, decreasing tension-mismatch is an important factor for the prevention of postoperative shoulder pain and stiffness.

Huberty et al^7^ reported 13.5% incidence of postoperative stiffness after transtendon repair, with 5% of patients requiring surgery for lysis of adhesions. Castagna et al^2^ concluded that shoulder discomfort after the transtendon repair technique is mainly due to tendon retraction.

Our approach is unique in treating PASR lesions with biceps augmentation for revision of articular-sided partial tears. Transtendon suture bridge repair biomechanically preserves intact bursal layers, restores anatomical footprints, and reduces tension mismatch.^6^^,^^8^^,^^18^^,^^24^^,^^25^ Revision repairs without tear completion are challenging due to poor tendon quality, adhesions, and compromised vascularity; however, biceps augmentation offers a collagen matrix, potentially enhancing the healing environment, even in massive cuff tears.^5^^,^^10^^,^^14^^,^^23^^,^^28^ After revision surgery using the biceps tendon augmentation, our patients have experienced decreased anterior shoulder pain. Long-term follow-up is needed to validate these outcomes, but our midterm results are promising with no serious complications.

### Limitations

First, a control group treated with tear completion for PASR lesions is needed for comparative analysis. However, midterm outcomes of our study showed the optimal treatment options (preservation of normal footprint and sufficient tendon thickness) for PASR lesion. We have observed an increase in PASR lesions after primary suture bridge repair of full-thickness tears, potentially expanding indications for biceps tendon revision repair. A primary limitation of this study is its relatively small sample size (N = 22). Additionally, this technique requires a learning curve compared to conventional tear completion and repair. Even though tear completion and cuff repair technique is technically easy, débridement or removal of retorn tendon with poor quality caused larger cuff defect and it made rotator cuff repair more difficult.

## Conclusion

PASR lesions are a rare entity, and their poor tendon quality complicates revision repair. Without tear completion, arthroscopic revision rotator cuff repair with biceps tendon augmentation (transtendon suture bridge repair with biceps tendon augmentation) is a reliable novel procedure, yielding improved functional and radiologic outcomes at midterm follow-up.

## Disclaimers

Funding: No funding was disclosed by the authors.

Conflicts of interest: The authors, their immediate families, and any research foundations with which they are affiliated have not received any financial payments or other benefits from any commercial entity related to the subject of this article.
